# Strength and Vulnerability: A Qualitative Study of Mental Health and Unmitigated Communion Among Female Migrants in Southeast England

**DOI:** 10.3390/ijerph23030330

**Published:** 2026-03-06

**Authors:** Patrick Nyikavaranda, Christina J. Jones, Marija Pantelic, Esohe Linda Abumwenre, Juliet Batista, Lijuan Wang, Mebrak Ghebreweldi, Tacye Turner, Priyamvada Paudyal, Dafni Katsampa, Carrie D. Llewellyn

**Affiliations:** 1 Division of Psychiatry, University College London, London W1T 7NF, UK; 2 Department of Primary Care and Public Health, Brighton & Sussex Medical School, University of Sussex, Watson Building, Falmer, Brighton BN1 9PH, UK; m.pantelic@bsms.ac.uk (M.P.); c.d.llewellyn@bsms.ac.uk (C.D.L.); 3 School of Psychology, Faculty of Health and Medical Sciences, University of Surrey, Guildford GU2 7XH, UK; c.j.jones@surrey.ac.uk; 4 Diversity Resource International, 36 St Nicholas Ln, Lewes BN7 2JZ, UK; 5 ICN Business School Artem, 86 rue du Sergent Blandan, 54003 Cedex Nancy, France; 6 Institute for Global Health and Wellbeing, School of Medicine, Keele University, Keele ST5 5BG, UK; p.paudyal@keele.ac.uk; 7 School of Life and Medical Sciences, University of Hertfordshire, Hatfield AL10 9AB, UK; 8 Cambridgeshire and Peterborough NHS Foundation Trust, Cambridge CB21 5EF, UK

**Keywords:** health inequity, migrant women, mental health, unmitigated communion, gender, social determinants, co-production

## Abstract

**Highlights:**

**Public health relevance—How does this work relate to a public health issue?**

- Mental health inequities among female migrants are shaped not only by service barriers but by gendered expectations of care and self-sacrifice embedded within migration contexts.
- Unmitigated communion functions as a psychosocial pathway through which social determinants of health become embodied as mental distress.

**Public health significance—Why is this work of significance to public health?**

- The study reframes unmitigated communion from an individual vulnerability to a socially patterned determinant of mental health inequity among migrant women.
- Co-produced qualitative evidence reveals forms of distress that remain largely invisible within conventional public health surveillance and service models.

**Public health implications—What are the key implications or messages for practitioners, policy makers and/or re-searchers in public health?**

- Public health and mental health services must recognise gendered caregiving and emotional labour as determinants of health when designing equitable, migrant-inclusive interventions.
- Equity-oriented co-production frameworks such as EMBaRK (Engaging Marginalised Communities by Building Relationships and Knowledge) offer scalable approaches for engaging marginalised populations and informing inclusive public health policy and research.

**Abstract:**

Unmitigated communion (UC), the prioritisation of others’ needs over one’s own well-being, is a critical lens for understanding the mental health of female migrants. This qualitative study explores how UC intersects with constructions of strength and vulnerability within this population, particularly amid challenges such as adaptation, discrimination, and gendered roles. Using a feminist participatory methodology, the study was co-produced with 10 migrant women and three professionals. Semi-structured interviews were conducted with 18 female migrants from 13 countries, representing diverse languages, cultures, and lengths of stay in the UK. Data were thematically analysed using the Engaging Marginalised Communities by Building Relationships and Knowledge (EMBaRK) framework, which centres lived experience and equitable collaboration. Through this analytic process, three key themes were generated: (1) perceived strength and resilience shaped by societal pressures and internalised self-reliance; (2) gender roles and self-sacrifice, including traditional caregiving expectations and neglect of personal health; and (3) isolation and reluctance to seek support, marked by concealed mental health struggles and stigma. Participants’ narratives revealed shared tensions between resilience and vulnerability. The findings highlight the central role of unmitigated communion in shaping migrant women’s mental health and underscore the need for gender-responsive, culturally informed interventions that support women to balance caregiving with self-care.

## 1. Introduction

“You are a fighter woman, and I know you’re going to achieve all your dreams and goals”

(Sarah, São Tomé and Príncipe).

Psychologist David Bakan introduced the concept of communion, a desire for connection and cooperation, in 1966 [1]. Traditional gender roles have often associated men with agency, characterised by self-assertion and independence, and women with communion, emphasising connection and care for others [2]. Contemporary scholarship, however, increasingly challenges such binary framings, recognising the fluidity and diversity of gender expression [3,4,5]. Although empathy and support are fundamental to social relationships, a persistent and unmitigated prioritisation of others’ needs over one’s own may have detrimental consequences [6]. This pattern is termed unmitigated communion (UC) [7]. Characterised by self-neglect and personal sacrifice, UC can disproportionally affect vulnerable groups, as it compels individuals to become overly invested in solving others’ problems at the expense of their well-being [8]. The significance of understanding the female migrant experience within the context of UC lies in the intersection of gendered expectations and migrant challenges, which profoundly impact mental health outcomes [9,10,11].

## 2. Literature Review

Societal norms frequently pressure women to prioritise their families and communal responsibilities, affecting various areas, including employment and a sense of belonging [12,13,14]. The strength and context of these impacts are significantly modulated by cultural, legal, and economic contexts and vary across different age groups, suggesting a complex interplay of demographic and societal factors [12,13,14,15]. Jayachandran discusses how gender norms influence the relationship between economic development and female employment, highlighting that cultural norms help explain significant differences in female employment among countries at the same level of development [15].

Moreover, the complexities associated with migration, including cultural adaptation, oppression, discrimination, and barriers to accessing essential resources, further complicate the multifaceted social and political pressures women already face [11,16,17,18,19]. Intersectionality, a critical concept introduced by legal scholar Kimberlé Crenshaw, recognises that individuals can experience overlapping and mutually reinforcing forms of discrimination based on their multiple social identities, such as gender, race, migrant status, and socioeconomic background [20]. Mora and Piper explore gender and migration from an intersectional and global perspective, emphasising that migration experiences are shaped by gender, class, race, and nation [21].

Building on the interchange of societal norms and their impacts, the exploration of how these dynamics are further complicated by migration is crucial. The intersection of gender and migration introduces unique risks and vulnerabilities in origin and destination contexts—vulnerability, as critically examined by Gilodi et al. [22], is deeply embedded within structural inequalities and societal norms that migrants encounter.

Survivors of catastrophic events exhibit markedly higher occurrences of mental disorders, with women notably experiencing increased rates of depression, post-traumatic stress disorder (PTSD), anxiety, and suicidal attempts compared to men [23,24,25,26]. This underscores the profound impact of gender on the mental health outcomes of disaster survivors [23]. Moreover, a broad spectrum of literature examines the vulnerability of various migrant groups at different stages of the migration journey [27,28,29,30]. A study by Tan and Kuschminder underlines the prevalence of gender-based violence (GBV) in particularly susceptible migrant groups, including forced and irregular migrants [27]. Consequently, it becomes clear that vulnerability cannot be considered purely innate or situational; a more profound understanding must encompass a broader array of factors beyond gender and sex, requiring a multidimensional, structurally informed approach within migration studies.

Furthermore, the role of gender in migration contexts reveals additional layers of complexity. Policies that tie a female migrant’s legal status to her employer can exacerbate her dependence on others, restricting her autonomy and making her more vulnerable to GBV [31,32]. Fear of losing her job and facing deportation often compels female migrants to endure abusive situations silently. This lack of agency not only perpetuates physical and emotional harm but also reinforces patterns of self-sacrifice and prioritisation of others’ needs over their well-being. These systemic inequalities amplify the conditions under which UC becomes a dominant coping mechanism. For many female migrants, the overwhelming cultural and societal pressures to “be strong” and provide for their families often translate into neglect of their own mental and physical health [13,33]. By failing to address the intersection of gendered, legal, and cultural vulnerabilities, such policies indirectly foster reliance on UC as a survival strategy. These gaps in cultural understanding and policy implementation thus contribute to heightened vulnerabilities for female migrants, underscoring the importance of addressing UC within the broader framework of migration policies [28].

Studies have consistently demonstrated the negative impact UC can have on the individual that can be detrimental to mental well-being, leading to symptoms of depression, anxiety, and self-esteem issues [2,34,35,36,37]. For female migrants already facing significant stressors, the combined effect of unaddressed mental health needs and the pressure to prioritise others can exacerbate their vulnerability and reluctance to access support [25]. Therefore, exploring UC in this population is essential for understanding how gendered and cultural expectations structure help-seeking and self-care, with direct implications for the design of responsive support services.

This study adopts an intersectional framework, recognising the complex and interconnected nature of social identities such as gender, migration status, race, and class [20]. Intersectionality draws attention to how multiple, overlapping vulnerabilities converge to produce health inequalities among migrants, and how these are shaped by broader social structures and contexts, including country of birth, ethnicity, social position, and conditions encountered across different phases of migration and settlement [38]. This framework enables an examination of how such intersecting factors shape the lived experiences and mental health challenges of female migrants [21].

In line with calls to move beyond conventional theoretical frameworks in migration and health research [38], this study additionally uses unmitigated communion (UC) as a conceptual and interpretive lens rather than as a standalone theoretical framework. UC informs the analytical orientation of the study by directing attention to how practices such as self-sacrifice, caregiving, emotional labour, and the prioritisation of others’ needs are articulated, negotiated, or resisted within participants’ accounts, without presupposing their form or meaning. Used alongside an intersectional perspective, this approach foregrounds how gendered expectations interact with migration-related, cultural, and structural factors to shape experiences of mental health. Together, these analytic lenses support an inductive examination of participants’ narratives that centres subjective meaning while remaining attentive to the broader social and structural contexts in which these meanings are produced.

In migration contexts, UC often arises as a response to gendered and cultural expectations, where caregiving and self-sacrifice are deeply rooted in societal norms [30]. For female migrants navigating the intersecting pressures of resettlement, economic survival, and familial obligations, UC can serve as both a coping strategy and a source of emotional strain, contributing to mental health challenges. This study investigates how female migrants reconcile these expectations while striving to prioritise their well-being and mental health.

## 3. Methodology

### 3.1. Study Design and Settings

This study forms part of a broader project exploring the mental health experiences and perceptions of female migrants residing in the Kent, Surrey, and Sussex (KSS) regions of Southeast England. A qualitative design informed by a feminist participatory action research framework was employed. This approach prioritised inclusivity and participants’ active involvement in shaping the research process. The study was conducted in two phases. In the first phase, the Engaging Marginalised Communities by Building Relationships and Knowledge (EMBaRK) framework for active involvement was co-developed with female migrants and professionals. See Supplementary File S1 for a detailed explanation of the EMBaRK framework. In the second phase, semi-structured interviews were conducted to explore participants’ lived experiences of unmitigated communion and mental health. A subset of interviews was co-facilitated by trained female migrant lived-experience researchers from the Female Migrant Co-Production Group, working alongside the academic researchers, reflecting the study’s feminist participatory design and the EMBaRK framework

### 3.2. EMBaRK: A Framework for Active Involvement

To ensure a more inclusive and representative approach to research involving female migrants, this study adopted the EMBaRK framework, a novel methodology developed specifically for this project. EMBaRK integrates co-production principles with feminist influences, emphasising the importance of including women’s voices and expertise throughout the process. This framework departs from traditional academic-led research paradigms by foregrounding the perspectives, experiences, and stories of female migrants, empowering them to guide every stage of the research process.

Beyond being a methodological tool, EMBaRK also served as an ethical framework that authentically reflected the perspectives and needs of female migrant participants, whether they migrated voluntarily or involuntarily (e.g., economic migrants, refugees, or asylum seekers). The process is symbolically envisioned as a collaborative boat journey, in which researchers, female migrants, and practitioners share responsibility for navigating the complexities of migration and mental health (see Supplementary Information). This metaphor underscores the collective effort and shared decision-making integral to the EMBaRK process.

#### Implementation of EMBaRK in Research Design

- The EMBaRK framework was operationalised through the formation of a Female Migrant Co-production Group (FMCG), comprising 10 female migrants and 3 professionals, including social workers and mental health advocates. This group played a central role in shaping the study procedures and ensuring the research authentically represented the lived experiences of female migrants. Specific contributions included:
- Research Priorities: Co-developing research questions to ensure relevance to female migrants’ lived experiences.
- Methodological Refinement: Providing feedback on the gender-responsive and culturally appropriate development of research tools, including interview guides.
- Data Collection Strategies: Advising on recruitment strategies and using interpreters to ensure gendered, linguistic and cultural accessibility.
- Participant Support: Advocating for measures to create safe and supportive environments during interviews, including providing well-being resources and referrals if needed.

These activities fostered a research environment rooted in trust, equality, mutual respect, and transparency. The collaborative process promoted a sense of ownership, empowerment, and relevance among group members, ensuring that the study authentically reflected the diverse contexts and experiences of female migrants.

In practice, EMBaRK informed key study procedures as follows:

1. Recruitment: Recruitment strategies were co-developed with the FMCG, focusing on reaching participants through trusted community organisations, social networks, and targeted campaigns. FMCG members reviewed and refined recruitment materials to ensure gender responsiveness, cultural sensitivity, and clarity. Linguistic accessibility was prioritised through the use of interpreters and bilingual advocates from community organisations.
2. Interviews: Semi-structured interview guides were co-produced with FMCG members, who provided iterative feedback to ensure relevance and sensitivity to participants’ gendered and cultural contexts. The EMBaRK principles prioritised participant agency during interviews, allowing participants to steer discussions toward the most relevant issues. Safe and supportive interview environments were created, with practical measures such as offering breaks, addressing emotional well-being, and providing follow-up referrals for support where needed.
3. Analysis and Write-Up: The EMBaRK framework guided data analysis to foreground participants’ voices. Preliminary themes were developed and reviewed collaboratively with the FMCG, who validated interpretations and provided critical feedback. Their input shaped how findings were framed, highlighting the intersection of unmitigated communion, cultural norms, and mental health. The final manuscript underwent FMCG review to ensure it accurately and respectfully represented participants’ perspectives.

### 3.3. Participants and Sampling

Participants were recruited through purposive and snowball sampling. Purposive sampling was used to identify female migrants who met the inclusion criteria; snowball sampling was then used to achieve broader reach through participant referrals. This approach aimed to gather sufficient data to address the research aims and support robust theme development through reflexive thematic analysis [39]. The strategy also reflected a commitment to diversity across migration trajectories, countries of origin, language, and mental health needs, prioritising depth and rich insight over representativeness in exploring UC.

### 3.4. Inclusion Criteria

Participants were self-identified female migrants aged 18 or older who currently lived in, or had lived in, the Kent, Surrey, and Sussex (KSS) region within the past five years. The term “female migrant” was used in recruitment materials to reflect the study’s focus and align with public and policy discourses. However, we recognise its limitations and potential exclusions. During interviews, participants were encouraged to describe their identities and migration experiences in their own terms.

Prior or current experiences of psychological distress or support needs were not inclusion criteria. This decision, developed in collaboration with the FMCG, acknowledged that mental health is articulated and experienced differently across cultural contexts. Including only those with a formal diagnosis or service use could have excluded individuals who experienced distress without recognising it as such, or who were unfamiliar with mental health terminology due to stigma or structural inaccessibility.

### 3.5. Recruitment Strategy

The Recruitment was conducted in two phases. In Phase 1, we contacted organisations supporting migrant communities across the KSS region, including Diversity Resource International (DRI), Ethnic Minorities in Canterbury (EMIC), Kent Refugee Action Network (KRAN), and the Refugee Buddy Project (RBP). Gatekeepers from these organisations were invited to circulate study information, including participant information sheets and adverts, through their networks. In some cases, such as with DRI, staff facilitated introductions between interested individuals and the research team. However, gatekeepers were not involved in participant selection, and all potential participants were allowed to contact the lead researcher directly. We also shared study materials on social media platforms, including X (formerly Twitter), and presented the study at relevant community events and conferences.

In Phase 2, we employed snowball sampling, whereby initial participants shared study information with others in their networks. This approach enabled us to reach women who may not have been engaged with formal services or digital platforms. All participants received a detailed information sheet and were invited to ask questions before giving informed consent. Eligibility was confirmed based on the inclusion criteria, and participation was entirely voluntary.

### 3.6. Procedure

#### Development of Topic Guide

The core research team, comprising academic researchers, the FMCG, and community-led organisations, co-produced a topic guide to ensure it addressed relevant topics and questions for female migrants. The broader Female Migrants’ Inclusive Network (FEMIN), which included female migrants, support groups, and academics, provided guidance and feedback throughout the development process. This collaborative, iterative approach ensured the guide was culturally sensitive, gender-responsive, and appropriate for the target population.

### 3.7. Data Collection

The lead researcher emailed or posted the participant information sheet, consent form, study advert, and demographic monitoring sheet to interested individuals, depending on their preference. Participants were encouraged to review the documents and ask questions before consenting. To reduce language barriers and promote inclusion, particularly for participants who might otherwise have been excluded due to limited English proficiency, support was offered from trained interpreters (Vandu Languages) and bilingual advocates from Diversity Resource International. Language needs were assessed during initial contact based on self-disclosure or communication preferences. Where required, interpreters or advocates assisted during the consent process and interviews.

Interviews were conducted in person or online using participant-preferred platforms such as Zoom or Microsoft Teams. Interviews typically lasted between 45–90 min and were flexibly paced according to participants’ preferences, comfort, and needs. The lead researcher audio-recorded and transcribed all interviews verbatim, ensuring adherence to ethical research practices and participant confidentiality.

### 3.8. Analysis

Transcripts were analysed using an inductive approach guided by Braun and Clarke’s framework for reflective thematic analysis [39,40,41]. Analysis began during the data collection phase, with transcription and preliminary familiarisation after each interview. This allowed for early identification of patterned meaning and informed ongoing reflection within the research and co-production groups.

The lead researcher developed initial codes and themes and iteratively refined them in collaboration with the FMCG, following the EMBaRK process. This dynamic and participatory approach allowed for responsiveness to variation within the sample and sensitivity to underrepresented voices. Reflexivity was embedded throughout the analysis, aligned with the feminist participatory action model, and supported critical engagement with assumptions, positionalities, and power dynamics.

The goal was to construct themes that identify and examine the underlying ideas, assumptions, conceptualisations, and ideologies that are theorised as shaping or informing the semantic content of the data ([40] p. 84).

In line with ethical research practice, all transcripts and interview data were anonymised, and pseudonyms were used throughout to ensure participant confidentiality.

### 3.9. Ethical Considerations

The study adhered to ethical research standards throughout all stages. Ethical approval was obtained from Brighton & Sussex Medical School (Ref: ER/BSMS9K87/1). All participants provided informed consent before participating, including permission to audio-record and transcribe interviews. Participants were informed of their right to withdraw from the study at any time without consequences. Confidentiality was ensured by anonymising transcripts and storing data securely in compliance with data protection regulations.

Participants received a £20 shopping voucher in recognition of their time and contribution, in line with ethical guidance on the involvement of community participants [42]. Vouchers were provided either electronically or in physical form, according to participant preference.

Measures were taken to support participants’ well-being during and after the study. Participants were provided with information about local mental health resources and support services. Interviews were conducted within a gender and culturally secure research environment, understood here as an approach that prioritises psychological safety, reflexivity, and attention to power dynamics related to gender, migration status, and language. In practice, this involved adopting a stance of cultural curiosity rather than assumption, allowing participants to define what felt relevant or sensitive within their own contexts, pacing interviews according to their needs, and remaining responsive to any signs of distress. Participants selected their own pseudonyms for the study, with guidance provided to ensure anonymity and avoid identifiable names. Interpreters were used when necessary to facilitate communication. The participatory approach, guided by the EMBaRK framework, prioritised transparency, mutual respect, and participants’ empowerment throughout the research process.

## 4. Results

### 4.1. Participant Characteristics

A total of 18 non-British-born women participated in the study, representing diverse countries of origin, including Turkey, Hong Kong, São Tomé and Príncipe, Palestine, Lebanon, Brazil, Ghana, Zimbabwe, Iraq, Ivory Coast, Uganda, Nigeria, and Portugal. The participants’ ages ranged from 24 to 59 years.

The sample reflected considerable ethnic, linguistic, and religious diversity. The majority were Black African (*n* = 7), followed by Arabic (*n* = 3). Portuguese (*n* = 5) was the most common first language, followed by Arabic and English (*n* = 3 each). Most participants resided in Sussex (*n* = 11), with others based in Kent or Surrey. Regarding religious affiliation, general Christians (*n* = 7) and Christian Catholics (*n* = 4) were the most represented groups. All participants identified as heterosexual. Over half reported having experienced or currently experiencing mood-related difficulties, such as depression, anxiety, or PTSD.

No participants withdrew from the study; all completed the consent process and interviews. The final sample size aligned with our expectations for achieving thematic saturation and capturing diverse lived experiences relevant to the research aims. See Table 1 for a summary of participant demographic and background characteristics.

### 4.2. Thematic Insights

Three key themes were developed concerning unmitigated communion (UC) and its significant impact on participants’ mental health and well-being. The first theme, perceived strength and resilience, highlighted how societal expectations and internalised self-reliance influenced participants’ coping mechanisms and sense of identity. The second theme, gender roles, societal expectations, and self-sacrifice, underscored the pressures participants faced from caregiving responsibilities, cultural norms, and the prioritisation of others’ needs over their own well-being. The third theme, isolation and reluctance to seek support, revealed participants’ struggles with loneliness, the concealment of mental health challenges, and limited access to supportive networks.

See Table 2 for a summary of these themes, including sub-themes and illustrative examples demonstrating how they contribute to UC’s complex and multifaceted experiences among female migrants.

Participant names have been changed to preserve anonymity. Verbatim quotations illustrate each theme and ground the analysis in participants’ lived experiences.

### 4.3. Perceived Strength and Resilience

Perceived strength and resilience emerged as a dominant feature of participants’ accounts, shaped by societal expectations to appear strong and self-reliant and influencing how women understood themselves and were perceived by others.

#### 4.3.1. Societal Expectations

Participants frequently described a perceived expectation to embody strength and resilience, shaping how they viewed themselves and how they believed others perceived them. The pressure to embody and conform to the “strong female migrant” identity was often experienced as externally imposed, circulating through family expectations, community norms, and broader societal narratives about migration and endurance. While this framing valorised survival and perseverance, participants described it as coming at a significant cost to their mental health. This internal and external pressure is captured in Gugu’s response, where she emphasises an expectation from herself and society:

“The concept of you have to just be strong and get on with life”

(Gugu, Zimbabwe).

Ndri encapsulates this sentiment, emphasising her role as the principal provider for her family, both in the UK and back in the Ivory Coast. She articulates the necessity of resilience in the face of their precarious circumstances: “You have to be strong. You have to be strong for the family” (Ndri, Ivory Coast). Her words highlight the relentless pressure she feels to survive and to be a source of strength for those who depend on her. Sarah also affirms it as a need to be strong and almost uses the word as a mantra to face the day:

“I need to be strong. That’s what I do every single day—to be strong”

(Sarah, São Tomé, and Príncipe).

Implicit societal expectations often shape how these female migrants are perceived as mentally strong. Several participants felt compelled to project an image of being both physically and emotionally tough, even when silently experiencing distress. This expectation was described as part of a broader social narrative that associates migration with strength and resilience, one that can obscure hidden vulnerabilities and discourage help-seeking:

“[As a migrant female] you have to be strong… and if you come and say you’re struggling with your mental health, you’re seen as weak”

(Obenewaa, Ghana).

Across these accounts, societal expectations regulated women, urging them to appear strong emotionally and physically despite distress. This framing hid vulnerability, limited help-seeking, and reinforced that endurance was expected and morally valued by the society.

#### 4.3.2. Internalised Mindset of Resilience

Participants described an internalised resilience mindset, viewing mental well-being as personal responsibility. Distress was seen as something to manage privately, through self-discipline or coping strategies, rather than as a reason for formal support. Some believed improvement depended mainly on personal effort, with Sarah stressing accountability for mental health outcomes:

“Even if you go to the doctor, if you do not help yourself… nobody is going to help you.”

“I think your support is you. Because if you get depressed, it’s because you want to be depressed”

(Sarah, São Tomé, and Príncipe).

Sarah underscores the importance of taking personal responsibility for one’s mental health. She posits that cultivating inner strength is a continual endeavour and asserts that individuals need to be accountable for their well-being.

While this perspective demonstrated a sense of agency and self-determination, it also highlighted a tendency toward self-blame, especially when distress persisted. Similar accounts appeared throughout the sample, with participants attributing barriers to support, when resilience failed, primarily to internal factors rather than systemic or institutional limitations. For instance, Elizabeth described internal exhaustion as the main obstacle to accessing assistance:

“The barrier is me. I haven’t got the strength to keep chasing after my support, so I’ve got so many other things I do during the day”

(Elizabeth, Portugal).

In this context, resilience was not simply a protective resource but also a mechanism through which responsibility for coping was internalised, often obscuring structural or relational constraints. Participants frequently described adopting self-care practices that prioritised distraction, endurance, or emotional containment rather than disclosure. Francesca explained how she relied on solitary creative activities recommended by her doctor to manage distress:

“My doctors suggested that I paint, as this is something that I enjoyed in the past. I now paint pictures at home, which makes me feel better”

(Francesca, Brazil).

Others framed resilience in terms of routine, productivity, or caring for others. Elizabeth described deriving strength from helping patients in her role as a care assistant, locating her sense of well-being in usefulness to others:

“I work as a house care assistant, and I help patients, and that makes me so satisfied… because I’m able to help these patients”

(Elizabeth, Portugal).

While these strategies offered moments of relief or meaning, they also tended to reinforce a pattern in which emotional needs were managed privately and seeking support was often delayed or deprioritised. For several participants, recognising vulnerability conflicted with deeply held beliefs about strength, independence, and self-sufficiency. Rachel reflected on this ambivalence, acknowledging her own hesitation to seek help while also perceiving it as a challenge to her self-image:

“Maybe there is something about me as well… my willingness to seek help, but it doesn’t help me to pick up the phone and talk to those professionals”

(Rachel, Turkey).

Together, these accounts demonstrate how an internalised resilience mindset can act as a double-edged sword. While it supports coping and perseverance, it can also sustain unspoken bonds by viewing mental distress as a personal failing, thereby reinforcing silence, self-monitoring, and delayed help-seeking.

### 4.4. Gender Roles and Self-Sacrifice

The intertwining of traditional gender roles and societal expectations significantly influenced the participants’ experiences of UC.

#### 4.4.1. Traditional Gender Roles and Cultural Norms

Many women felt a strong sense of duty to prioritise the needs of their families and communities, often at the expense of their own mental and physical health. This was particularly evident in the narratives of participants like Ndri from the Ivory Coast, who felt pressured to downplay her menopausal symptoms due to the expectation that women should always be strong and available for others. Ndri stated,

“It is part of being a woman. They can’t be agreeing to every request [to take time off due to menopausal symptoms] all the time. I am sure men have their problems, too. Even if I am at home, I still have to live with the symptoms outside of work [and look after my family]”

(Ndri, Ivory Coast).

Furthermore, Ndri, who self-identified as the primary provider and caregiver in her household, astutely observed that even in households with traditional mother–father structures, women often bear disproportionate responsibility for meeting the family’s needs. In both her own experience and broader contexts, the expectation for females to always appear strong prevailed:

“It is only the wife taking care of the kids taking care of the house all that so you can’t be having mental health issues”

(Ndri, Ivory Coast).

This internalised pressure to be strong and resilient was further reinforced by cultural norms that emphasise women’s roles as caregivers and the normalisation mechanism of stoicism:

“In my country, in Ghana, everybody’s literally born frustrated… everybody’s going through a lot, but it’s not something that has a lot of focus… we just grow up thinking OK, this is normal and then you brush it under the rug and go about your day”

(Eelin, Ghana).

As Obenewaa from Ghana expressed, a woman seeking help for mental health struggles could be perceived as a sign of weakness, leading to feelings of shame and isolation. She mentioned:

“[As a migrant female] you have to be strong, whatever, and if you come on to say, oh, I’m struggling with my mental health because of AB and C, you [are] seen as weak or something like that”

(Obenewaa, Ghana).

#### 4.4.2. Neglect of Personal Health

The prioritisation of family well-being over personal health was a recurring theme, particularly among participants who had recently migrated due to conflict or economic hardship. Jawaria from Iraq, for example, described how she pushed herself to support her children despite her trauma and emotional distress:

“I was like trying to support my kids. You know, instead of looking after myself… ignoring I’m not feeling well, I’m not good. But I was pushing myself to support them”

(Jawaria, Iraq).

Similarly, Masiko from Uganda chose to continue working long hours to provide for her daughters back home, even though she was experiencing symptoms of depression and exhaustion. She shared,

“My girls are young. They need me. They need their mother in growing up. And I need to be there more for them. I need to be composed”

(Masiko, Uganda).

Neglect of personal health was not always experienced as a deliberate choice but often emerged through the gradual erosion of self-care in the context of sustained caregiving and responsibility. Participants described pushing through physical and emotional symptoms while prioritising the needs of others.

“You find it hard to get up, you can’t take care of yourself, you just wish you could give the kid to someone else and be just in your corner”

(Ndri, Ivory Coast).

An additional dimension emerged: a profound self-sacrifice, self-neglect, and overlooking one’s needs while tending to ailing family members. The weight of responsibility often eclipsed personal well-being, echoing caregivers’ silent struggles in their unwavering commitment to helping others.

“I became the carer…but you know, you forget about yourself and it’s all about the other person”

(Nakabiri, Uganda).

Sleep disturbance frequently functioned as an early indicator of distress, with participants describing insomnia, fatigue, and rumination that were normalised rather than recognised as signals of declining mental health:

“When I first came to the country I didn’t think I was depressed, I just felt I was sleepy. But upon reading about it later on, I realised that was like signs of depression”

(Eelin, Ghana).

Several participants, including Eelin, reflected on how psychological distress was only recognised retrospectively, with symptoms initially interpreted as ordinary tiredness or stress rather than as mental health concerns:

“I had no idea those were signs of depression, I just thought I was sleeping (for long periods), I just thought it was just normal”

(Eelin, Ghana).

For some participants, neglect of personal health was profoundly intertwined with experiences of institutional judgment and perceived discrimination, contributing to enduring patterns of distress. Francesca described how her mother’s physical symptoms were dismissed by a GP shortly before her death, an encounter she attributed to her mother’s migrant status:

“The day before my Mum passed away, she went to the GP in Spain and complained of pains in her head and chest… I think the doctor didn’t bother about her as she was a migrant, and he should have sent her to hospital”

(Francesca, Brazil).

Following her mother’s death, Francesca also described being subjected to explicit judgment and blame, including accusations that she had failed to care for her mother adequately:

“They said that I hadn’t looked after my Mum and was waiting for her to die, which deeply upset me as I did everything I could to care for and look after her”

(Francesca, Brazil).

Francesca proceeded to describe ongoing psychological distress, including insomnia, intrusive memories, cognitive struggles, and dependence on medication. Despite her declining mental health, she kept up with daily responsibilities in education, administration, and self-care. Her story shows how UC may persist beyond caregiving, manifesting as endurance, self-blame, and self-sacrifice, especially amid feelings of institutional rejection and unresolved trauma.

Participants consistently described their caregiving responsibilities as moral obligations, deeply tied to identity, family roles, and gendered-cultural expectations. Nakarabi and others viewed caring for relatives not as a burden, but as something they were expected to do, regardless of the toll on their health.

This sense of duty often led to self-sacrificing behaviours, including neglecting their physical and mental well-being. Many reported insufficient sleep, lack of rest, and delaying or forgoing care to support others. Several participants also expressed guilt about prioritising their needs, further compounding their emotional burden. Gugu from Zimbabwe reflected on this inner conflict, describing hesitation to seek support due to concerns about taking resources away from others:

“So even asking for help it’s like oh, am I being too weak and am I using resources that other people may need more of than myself, you know?”

(Gugu, Zimbabwe).

These narratives underscore how gendered expectations of caregiving, while often valued and framed as a strength, contributed to psychological strain through guilt, internalised expectations, and reluctance to seek support.

### 4.5. Isolation and Concealment

Many participants reported concealing their mental health struggles due to the stigma of mental health issues, lack of support, or to avoid worrying their families.

#### 4.5.1. Concealment of Mental Health Struggles

The concealment often contributed to feelings of isolation and loneliness. For Liberty, this meant withholding distress from relatives abroad:

“I wouldn’t tell my family in Hong Kong about my difficulties here. I don’t want to worry them”

(Liberty, Hong Kong).

However, concealment also reflected broader relational and social considerations. Some participants described how expectations placed on migrant women within families and communities shaped decisions to remain silent about mental health struggles, particularly where disclosure was perceived to carry reputational consequences. Nadia reflected:

“There was the stigma of oh, no… (the girls in the family) are mentally disabled now and we’re needing support… we’ll just keep this quiet. Because no one’s gonna marry anyone in this family”

(Nadia, Iraq).

This account illustrates how concealment is influenced not only by individual stigma but by concerns over family reputation and future social prospects. In Nadia’s narrative, silence functions as a protective strategy aimed at preserving family honour and managing how mental health is perceived within the wider community. In this context, concealment extends beyond emotional caretaking to encompass reputational labour, reinforcing a prioritisation of familial and social standing over individual well-being.

Not all participants’ reluctance to seek support was driven by stigma or lack of awareness. For some, concealment functioned as a deliberate strategy to manage emotional boundaries within family relationships. Ms Din described recognising her distress following the loss of her baby and subsequent breakdown of her marriage, yet choosing not to engage with formal support despite being offered access:

“I know why I’m sad… I don’t want to say it again and feel it again. I just want to get over it”

(Ms Din, Palestine).

Rather than reflecting denial, this account illustrates how emotional containment can operate as a form of relational care. Ms Din described actively limiting disclosure to protect her daughter from emotional burden, withdrawing from shared conversations after being advised that “negative things” might affect her child. In this context, concealment was not solely about stigma but about preserving family well-being, highlighting how UC may manifest through the management of others’ emotional safety at the expense of one’s own need for support.

#### 4.5.2. Feelings of Isolation and Loneliness

Some of the interviewees often feel compelled to conceal their emotional needs and mental health struggles to maintain a facade of strength in their family roles. The experience of going through these difficulties alone, without support or the ability to share emotions with loved ones, likely compounds the isolation and loneliness that many of the female migrants who were interviewed face. The “strong female migrant” narrative can perpetuate feelings of isolation and concealment of mental health struggles. While this archetype dominated the narratives of many participants, some individuals, like Jawaria, challenged expectations of stoicism and resilience. Despite initially appearing strong and capable, she revealed the emotional toll:

“I might look like I’m…standing and very strong, but I…don’t feel like I’m that strong, but as soon as something comes up, I feel like the first thing I’ll do is like give up”

(Jawaria, Iraq).

Jawaria’s confession exposes the complex interplay among gendered-cultural expectations, the internalised pressure to prioritise others, and individual vulnerabilities. Her words highlight the hidden loneliness and inner conflict that many female migrants may experience while maintaining an outward facade of strength.

For others like Rachel, concealment became internalised as a form of self-regulation. Rachel described masking her loneliness by normalising distress:

“I will feel very lonely. Yeah, so…I will tell myself that it was normal when in reality I feel it wasn’t normal”

(Rachel, Turkey).

This underscores the internal conflict female migrants might face between the need to appear strong and their hidden struggles.

## 5. Discussion

The findings of this study reveal the profound impact of unmitigated communion (UC) on the mental health and well-being of female migrants, highlighting the interplay between gender roles, cultural expectations and individual coping mechanisms. Thematic analysis identified critical dimensions of UC, including perceived strength and resilience, gender roles and societal expectations, self-sacrifice for family welfare, isolation, and a reluctance to seek support. These findings align with existing research on the significant mental health burdens faced by female migrants due to gendered expectations and socio-cultural pressures [15,43].

The notion of the ‘strong migrant woman’, circulating in policy, media, and sometimes within migrant communities themselves, creates a double bind: while it celebrates endurance, it can silence vulnerability. As Ichou and Wallace note, the “healthy migrant” effect, while statistically observed in some contexts, can obscure structural inequities and deflect attention from the lived realities of distress and exclusion [44]. In this study, participants’ accounts illustrate how such narratives can be internalised and reproduced, shaping expectations of self-reliance and discouraging help-seeking even in the face of significant emotional strain [45].

The theme of self-sacrifice for family well-being underscores a critical dimension of UC within this study. Participants described scenarios in which they routinely deprioritised their health in the context of caregiving responsibilities, reflecting patterns widely documented in migration and gender scholarship [46]. Existing research demonstrates that caregiving roles and familial expectations disproportionately burden female migrants, often exacerbating their mental and physical health challenges [47,48]. Feminist migration studies also emphasise that these responsibilities are negotiated in complex ways, influenced by both structural constraints and individual agency [49,50,51]. Such findings highlight the need for healthcare frameworks that recognise and address the unique pressures female migrants face. Additionally, fear of losing their caregiver role if they admit to mental health struggles can further perpetuate this cycle of self-sacrifice [25].

Beyond physical caregiving responsibilities, participants also bore the burden of cognitive labour; the ongoing mental tracking, organising, and emotional regulation involved in managing both their own lives and the needs of others. This form of invisible, gendered labour—encompassing planning, vigilance, and emotional responsibility—is well documented in feminist scholarship [52,53,54] and contributed to the exhaustion described by many participants. Studies have shown that women disproportionately undertake the cognitive and emotional dimensions of care work, including anticipatory planning, monitoring, and managing others’ feelings [54,55,56]. Such labour is frequently unrecognised yet contributes significantly to stress, time pressure, and emotional exhaustion [57], mirroring the experiences described by many participants in this study.

The pervasive stigma associated with mental health within migrant communities remains a significant barrier to accessing support [58,59,60,61]. Participants often concealed their mental health struggles due to shame, fears, and assumptions of mental illness being an individual factor. This theme illustrates that stigma functions not merely as a societal constraint but also as a critical determinant of health, deterring participants from recognising their distress, delaying help-seeking, and normalising psychological hardship as an individual responsibility. Many concealed their struggles out of fear of stigma or burdening their families. This reluctance to seek help, coupled with norms of self-reliance, reinforces unmitigated communion by normalising self-sacrifice and concealment, thereby intensifying mental health difficulties over time.

These interlocking processes of gendered caregiving, institutional judgement, and perceived discrimination were particularly evident in accounts where participants described being blamed or morally scrutinised following illness or bereavement, reinforcing patterns of endurance, concealment, and self-sacrifice.

By exploring culturally rooted coping strategies and the circumstances that support or limit them, this study sheds light on how female migrants manage the tension between their caregiving roles and their own well-being. Instead of viewing self-care as separate from caring for others, the findings highlight the importance of culturally and gender-sensitive approaches that reduce stigma and acknowledge caregiving as a core part of many female migrants’ identities. These approaches should also create space for vulnerability, support, and self-preservation.

### 5.1. Strengths and Limitations

Implementing the EMBaRK framework in this study offered several distinct strengths, particularly its innovative and participatory methodology. By adopting a co-production approach involving close collaboration with female migrants and community-led organisations, the study fostered trust and rapport with participants who might otherwise be hesitant to engage in academic research. This trust-building process was pivotal in authentically representing the voices and perspectives of female migrants, particularly those from marginalised communities.

This study is among the first to explore the concept of unmitigated communion (UC) among female migrants in the UK, addressing a critical gap in the literature. Combining the EMBaRK framework with a demographically diverse sample enabled a richly textured understanding of UC. The co-production of the topic guide, including the involvement of trained interpreters and linguistic accessibility, further enhanced the study’s cultural sensitivity and relevance.

While qualitative research does not seek statistical generalisability, certain limitations should be acknowledged. While spanning 24–59 years, the age range skewed toward mid-life women, potentially underrepresenting younger or older migrant voices. Additionally, although recruitment strategies aimed for broad diversity, most participants aligned with the UC narrative, and few offered strong counter-perspectives. This may reflect either the reality of shared experiences or a limitation in sample variation. Future studies should be attentive to this possibility. Furthermore, stigma surrounding mental health may have dissuaded some potential participants from taking part, potentially excluding perspectives of those least likely to disclose distress. Given that UC often operates beneath the surface of everyday interactions and that stigma may inhibit open disclosure, future research may benefit from more discreet, context-sensitive methods, such as ethnography or embedded participatory observation. Although thematic saturation was reached, these limitations should be considered when interpreting the findings.

### 5.2. Researcher Positionality and Reflexivity

The lead researcher, a male academic, recognised the inherent challenges of exploring gendered experiences of female migrants. This positionality shaped the reflexive approach adopted throughout the study. The EMBaRK framework, co-developed with the Female Migrant Co-production Group (FMCG), was instrumental in establishing trust, mutual respect, and collaborative power-sharing.

To mitigate potential biases and imbalances, the research team engaged in continuous reflection and iterative dialogue with the FMCG and FEMPAR (Female Migrant Participatory Action Research Network). This approach helped ensure that participants’ voices remained central to the research process and interpretation. The study upheld a commitment to methodological transparency and ethical integrity by embedding reflexivity and shared authorship into the analytical stages.

### 5.3. Recommendations for Research and Practice

UC often operates beneath the surface of everyday interactions, and innovative approaches, such as ethnographic methods or participatory action research, could offer deeper insights into how it manifests in various contexts. It would also be valuable to investigate how UC presents in different subgroups of female migrants, such as asylum seekers, versus those settled for more extended periods. Variations may exist based on settlement status, life stressors (e.g., housing insecurity, deportation fears), and length of time in the host country.

Expanding the scope of UC research to include male migrants, particularly refugees, could illuminate how societal expectations of masculinity and patriarchal roles related to providing and caregiving might influence their mental health and help-seeking behaviours. This could further refine our understanding of how gender intersects with cultural norms and migration experiences to shape mental health outcomes.

From a practical standpoint, the findings of this study have several implications. Understanding UC can enhance the assessment, formulation, and treatment of female migrants presenting with mental health concerns. Healthcare professionals should employ culturally and gender-secure approaches, actively seeking to understand how UC is conceptualised within different cultural and gendered contexts. This includes demonstrating curiosity about how migration has shaped the individual’s experience of UC and mental health. Targeted questions about mental health needs, cultural understanding of mental illness, and support networks can foster a therapeutic environment where female migrants feel understood and better able to access the help they need.

Intervention studies highlight the importance of culturally adapted care in addressing the unique challenges faced by migrants. For instance, group-based culturally tailored interventions, such as culturally adapted cognitive-behavioural therapy (CBT), have shown promising outcomes in reducing mental health stigma and improving access to care among ethnic minority women, including migrants [62]. Peer support models have similarly demonstrated the value of fostering social connection and belonging among female migrants, helping them to balance caregiving responsibilities with self-care. These findings underscore the potential for culturally specific interventions to mitigate the isolating effects of UC while promoting resilience.

Furthermore, clinicians can be crucial in signposting women to peer support groups and community resources that foster social connection and belonging. Recognising the isolating effects of UC, healthcare professionals can empower women to prioritise self-care and connect with others who share similar experiences. This could involve recommending social activities, befriending services, or culturally and gender-specific support groups that cater to the unique needs of female migrants. By addressing the social and emotional aspects of well-being alongside clinical interventions, healthcare providers can offer a more holistic and practical approach to supporting the mental health of female migrants.

Future research should explore the effectiveness of interventions explicitly designed to address UC in migrant populations, examining how such approaches can empower women to balance caregiving responsibilities with their mental and physical health needs. Investigating the scalability and cultural adaptability of existing models, such as CBT, may also provide valuable insights for tailoring support to diverse migrant communities.

## 6. Conclusions

The study highlights the complex interplay where societal expectations, sacrifice for others, and internalised strength and reluctance in help-seeking play significant roles in shaping the experiences and perceptions of mental health and support for newly arrived female migrants. The paradox in the current study paints a complex situation. While the female migrants interviewed in the current study felt compelled to work extra hours to care for their loved ones, their potential physical absence may inadvertently create an anomaly. Specifically, an unmitigated commitment to work to support loved ones becomes counterintuitive when it harms their mental and physical health, potentially leaving them unavailable to provide essential support during critical times. Further research is warranted to explore and understand this incongruity. Furthermore, these complex and intersecting experiences underscore the need to investigate and implement targeted, holistic approaches to supporting the well-being of female migrants.

The complex relationship between societal expectations, gender roles, and the silent burden of caregiving responsibilities has profound implications for the mental health of female migrants. This study not only highlights the need for targeted mental health interventions but also calls for comprehensive policy and clinical adjustments that consider the unique challenges faced by this demographic.

To enhance the depth of our findings, we have included a lived experience commentary from an FMCG member. This independent commentary provides valuable insights by observing and reflecting on the research process and the findings as a “critical friend”.

### Lived Experience Commentary

UC is a very complex concept. My understanding is that UC occurs when a female migrant attaches too much value or importance to ‘Communion’ (Caregiving relationships) and, therefore, neglects their personal mental health needs. The reasons why are very varied. However, this is complex because the ‘Communion’ relationships often give the female migrant a sense of purpose, joy, love and satisfaction, significantly improving their mental health. If these relationships were reduced or removed, this would negatively affect their mental health, as well as the fear and worry related to losing these relationships (JB).

## Figures and Tables

**Table 1:** Demographic and background characteristics of participants (*N* = 18).

Characteristics	Category	Number
Gender and sex	Self-identify as female/woman	18
Age	18–24	2
	25–34	4
	35–44	8
	45–50	1
	50+	3
Ethnicity	White European	1
	White Brazilian	2
	Asian—Hong Kong Chinese	1
	African Other	1
	Turkish	1
	Black European	1
	White—Other	1
	Arabic	3
	Black—African	7
First Language	Twi	1
	English	3
	French	1
	Turkish	1
	Luganda	2
	Ndebele	1
	Arabic	3
	Portuguese	5
	Cantonese	1
Religion	Christian	7
	Christian—Anglican	1
	Christian—Catholic	4
	Muslim	2
	Atheist	1
	No religion	1
	No recorded religion	1
Sexual Orientation	Heterosexual	18
Country of Origin	Turkey	1
	Hong Kong	1
	São Tomé and Príncipe	1
	Palestine	1
	Lebanon	1
	Brazil	3
	Ghana	2
	Zimbabwe	2
	Iraq	1
	Ivory Coast	1
	Uganda	2
	Nigeria	1
	Portugal	1
Interpreter required	Yes	6
	No	12
KSS County	Kent	4
	Surrey	3
	Sussex	11
Length of stay	<1 year	3
	1–5 years	7
	6–10 years	5
	>10 years	3
Difficulties with mental health	Mood Disorders (depression, anxiety, PTSD)	10
	Other (Including stress-related illnesses)	5
	Not stated	2

**Table 2:** Themes related to unmitigated communion and mental health in female migrants.

Main Theme	Sub-Themes	Explanatory Text
Perceived strength and resilience	Societal expectations	“Healthy migrant” phenomenon: Pressure to conform to the stereotype of being physically and mentally tough despite facing challenges.
	Internalised mindset of resilience	Belief in self-reliance and personal responsibility for mental well-being.
Gender roles and self-sacrifice	Traditional gender roles and cultural norms	Cultural norms that reinforce the caregiving role primarily for women. Pressure to downplay personal health issues to conform to these roles
	Neglect of personal health	Neglecting symptoms of illness or delaying seeking medical care. Insufficient sleep, rest, or relaxation due to prioritisation of caregiving responsibilities.
Isolation and reluctance to seek support	Concealment of mental health struggles	Fear of being seen as weak or a burden if they express mental health challenges. The stigma surrounding mental health within certain cultures or communities.
	Feelings of isolation and loneliness	Limited social support networks due to migration and language barriers. Difficulty connecting with others due to cultural differences or feelings of shame.

## Data Availability

The qualitative data generated and analysed during this study are not publicly available. The interview transcripts contain sensitive personal narratives and contextual information that could risk participant identification if shared. In line with the ethical approvals governing this study and the commitments made to participants regarding confidentiality, the data are therefore not suitable for public release.

## Supplementary Materials

The following supporting information can be downloaded at: https://www.mdpi.com/article/10.3390/ijerph23030330/s1, Supplementary File S1: The EMBaRK (Engaging Marginalised Communities by Building Relationships and Knowledge) Framework. Figure S1: Components of the EMBARK process. Figure S2: EMBaRK boat journey process.
