# Supplementary material for: Strength and Vulnerability: A Qualitative Study of Mental Health and Unmitigated Communion Among Female Migrants in Southeast England
Source: Int J Environ Res Public Health. 2026 Mar 6;23(3):330. doi: 10.3390/ijerph23030330 (PMC13027263; doi:10.3390/ijerph23030330)
Supplement: Supplementary file 1 [file ijerph-23-00330-s001.zip › ijerph-4134235-supplementary.pdf]

Supplementary File 1: The EMBaRK Framework

Principles of EMBaRK:

The EMBaRK framework is founded on six core principles:

1. **Trust and Relationship-Building:** Creating safe, inclusive spaces where participants feel valued and respected.
2. **Community Ownership and Leadership:** Empowering participants to actively lead and shape the research process.
3. **Reflexivity and Adaptability:** Ensuring researchers critically reflect on their assumptions and adapt processes to emerging insights.
4. **Inclusivity and Equity:** Centring diverse voices, particularly those from marginalised communities, to co-create knowledge.
5. **Dignity and Empowerment:** Recognising the intrinsic value of participants and fostering an environment of mutual respect.
6. **Collaboration and Sustainability:** Building lasting partnerships and ensuring knowledge generated remains impactful.

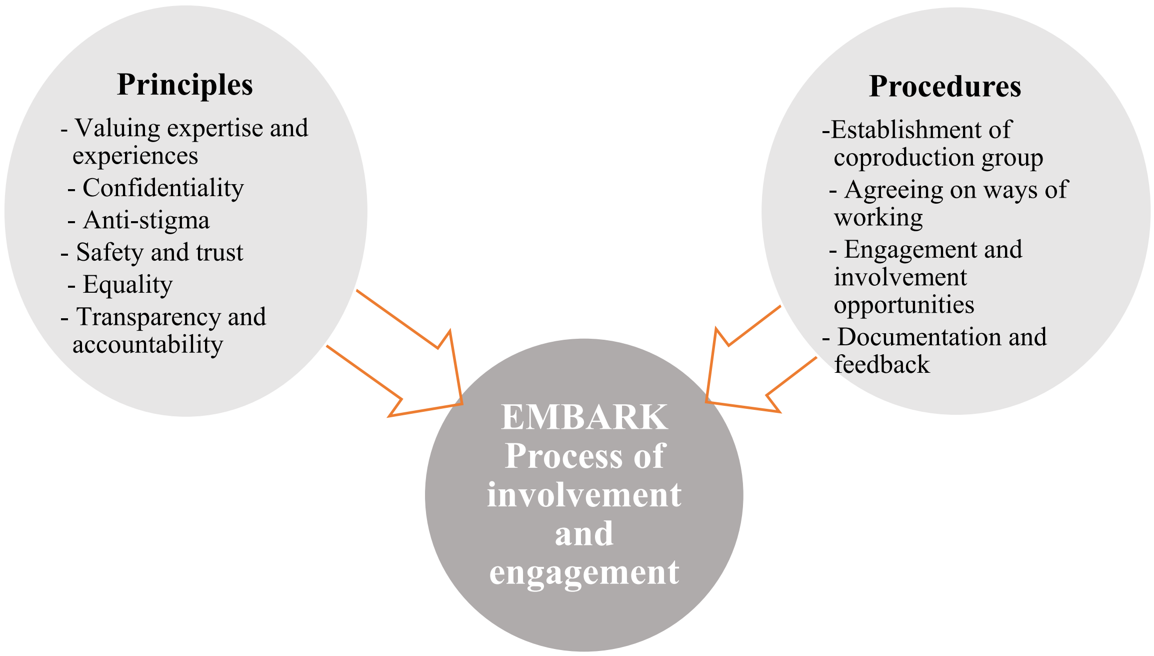

Figure 1: Components of the EMBARK process

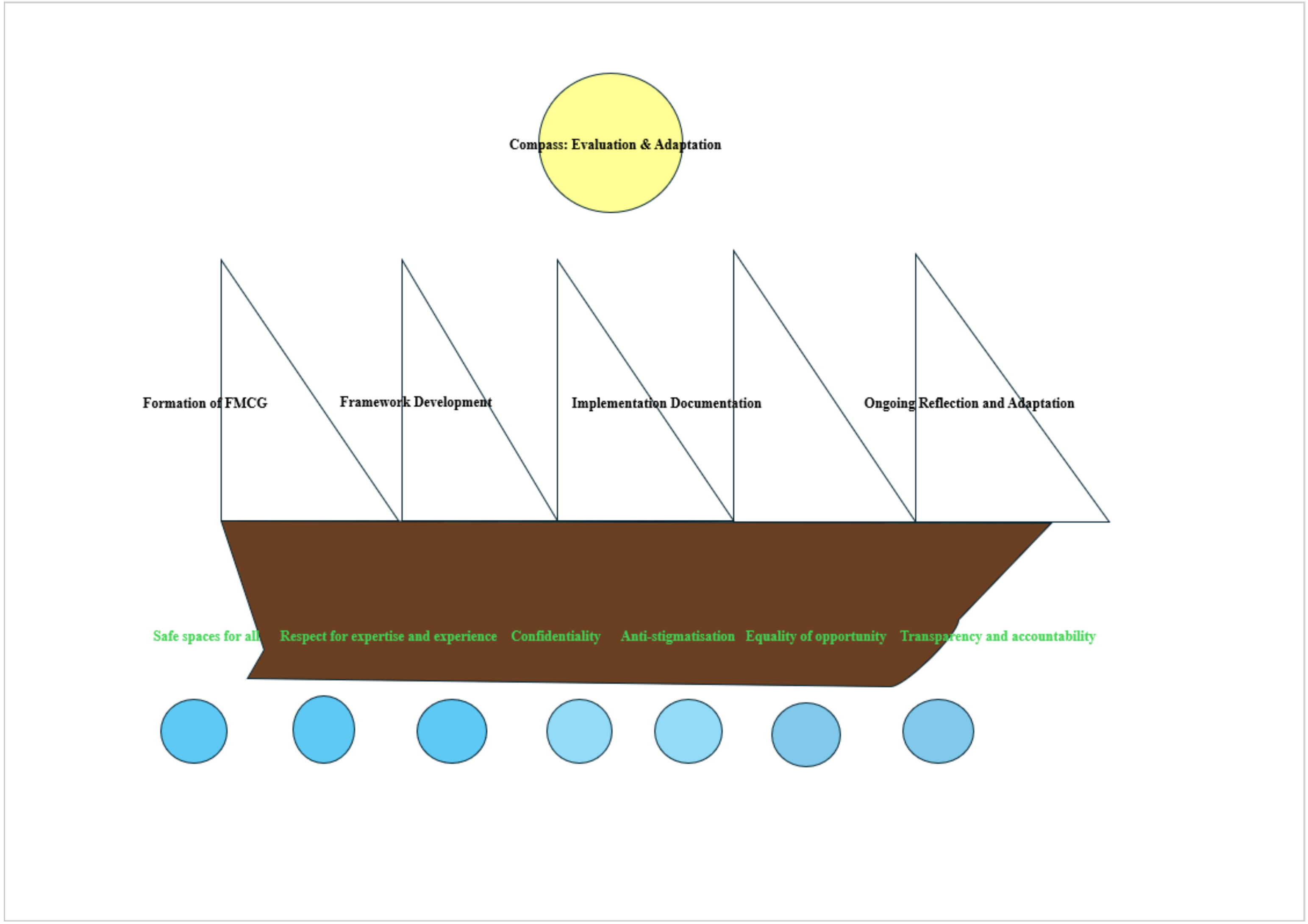

Figure 2: EMBaRK boat journey process

Implementation of EMBaRK:

The EMBaRK process was operationalised through the Female Migrant Co-Production Group (FMCG), comprising female migrants and professionals. The group co-produced research priorities, methods, and tools through a series of iterative feedback loops. Gender and cultural sensitivity and linguistic accessibility were prioritised through trained interpreters and community engagement.

The Boat Journey Metaphor:

The EMBaRK framework is symbolised as a rowing journey, where researchers and participants collaboratively navigate the challenges of research. Key components include:

- **Hull:** Trust and inclusivity, forming the foundation.
- **Oars:** Collective tasks and responsibilities that propel the research forward.
- **Compass:** Reflexivity and adaptability, guiding the process.
- **Provisions:** Resources, knowledge, and emotional support sustaining the journey.
- **Crew:** Researchers, participants, and stakeholders working collaboratively to achieve shared goals.

This metaphor highlights the resilience, trust, and shared effort required to navigate both research complexities and systemic inequities.

Flexibility and Adaptability:

The EMBaRK framework is designed to be versatile, with its acronym adapting to specific contexts. For example, in this study on female migrants, EMBaRK stands for *Empowering Migrant Bridges: Active Research and Knowledge*. Its flexibility ensures cultural relevance and participant ownership in any research setting.
